# Preparation and performance study of CTBN/nano-SiC composite toughened *Xanthoceras sorbifolia* oil-based adhesive

**DOI:** 10.1039/d5ra09970f

**Published:** 2026-05-14

**Authors:** Rao Duan, Jie Wang, Yihua Zhang, Zhenpeng Wang, Lei Jiao, Yan Long, Tao Hou, Gaole Zhao, Yinan Hao

**Affiliations:** a College of Material Science and Art Design, Inner Mongolia Agricultural University Hohhot 010018 China nanyihao83@163.com; b National Forestry and Grassland Engineering Technology Research Center for Efficient Development and Utilization of Sandy Shrubs Hohhot 010018 China; c Tianjin 729 Sports Goods Co., Ltd Tianjin 300392 China

## Abstract

Adhesives used in traditional plywood predominantly rely on petroleum-based materials, which pose issues of formaldehyde emission and present challenges in balancing the toughness and rigidity of the plywood. In this study, a base resin system was constructed using *Xanthoceras sorbifolia* oil as the raw material, by blending acrylated epoxidized *Xanthoceras sorbifolia* oil (AEXO) with *Xanthoceras sorbifolia* oil dimethacrylate (MXOEA), followed by crosslinking with 2-isocyanatoethyl methacrylate (IEM). Carboxyl-terminated butadiene-acrylonitrile rubber (CTBN) and nano-silicon carbide (SiC) were introduced for composite toughening, and the effects of their contents on the properties of the adhesive and the resulting plywood were investigated. The results indicated that the optimal formulation was achieved with 15% CTBN and 0.1% SiC: the impact strength of the adhesive increased by 142.7% compared with that of the unmodified system. The plywood prepared with this adhesive met the key mechanical and water-resistance requirements for high-performance wood-based composites; further processing it into table tennis blades verified the practical application potential of this adhesive. This study provides a green alternative to petroleum-based adhesives in the fields of plywood and wood-based sporting equipment.

## Abstract 2

Using *Xanthoceras sorbifolia* oil as a raw material, a high-performance adhesive was successfully prepared and applied in table tennis blade plywood, providing an ideal sustainable alternative to petroleum-based adhesives.

## Graphical abstract

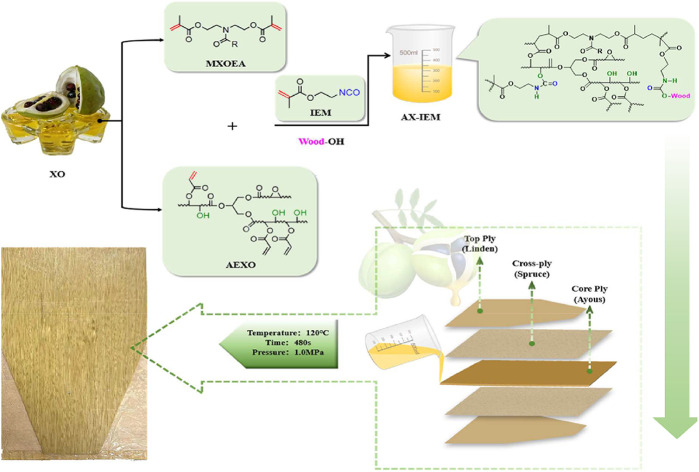

## 1. Introduction

As an important engineered wood-based composite, the performance of plywood largely depends on the properties of the adhesive used. In application fields such as sporting equipment, furniture, and decorative materials, where high mechanical performance and environmental friendliness are required, the structural integrity and environmental adaptability of plywood are particularly critical.^1^ Currently, the adhesives widely used in the plywood industry, such as phenolic resin, urea-formaldehyde resin, and epoxy resin, mainly rely on petrochemical resources and may release volatile hazardous substances such as formaldehyde during curing or service. Therefore, the development of formaldehyde-free adhesives based on renewable feedstocks that combine high performance with environmental friendliness has become an important research direction for achieving green and sustainable development in the field of wood-based composites.^2–4^

Existing bio-based adhesive feedstocks each have their own limitations. Tannin contains polyphenolic groups, and its bonding performance is similar to that of phenolic adhesives; it is currently mainly used for wood bonding.^5^ However, tannin-based adhesives are prone to decomposition at high temperatures, and the plywood prepared with them exhibits poor water resistance. The molecular structure of lignin contains active groups such as aromatic groups and phenolic hydroxyl groups;^6^ in the preparation of wood adhesives, lignin is often used as a substitute for phenol. However, due to the complexity of lignin molecules and the variability of their reactions, it is difficult to obtain the desired products. Sugar-based adhesives are rich in hydrophilic hydroxyl groups, and therefore commonly suffer from poor water resistance and susceptibility to mildew;^7^ hydrophilic biomass feedstocks such as soy protein generally face interfacial bottlenecks including chemical surface heterogeneity, strong hydrogen bonding networks, and hierarchical pore structures,^8^ resulting in limitations such as poor water resistance and insufficient bonding strength.^9,10^ In contrast, vegetable oils, owing to the hydrophobic nature of their fatty acid chains,^11,12^ have become ideal feedstocks for preparing water-resistant adhesives.^13^ Meanwhile, the presence of highly reactive sites in triglycerides,^14^ such as double bonds, allyl groups, and ester groups, enables them to undergo chemical modifications such as epoxidation and acrylation to produce high-performance polymers.^15^

Although some progress has been made in the research on vegetable oil-based adhesives in recent years, existing systems have mostly focused on common oil crops such as soybean oil and palm oil; research on adhesives derived from *Xanthoceras sorbifolia* oil, a high-value resource, remains unexplored. As a woody oil tree species, *Xanthoceras sorbifolia* produces oil with an unsaturated fatty acid content exceeding 90%, providing abundant C=C reaction sites for chemical modification. However, owing to the low reactivity of these C=C bonds, it is necessary to introduce reactive groups through acrylation and epoxidation reactions to construct high-performance polymers.^16^ Although the acrylated epoxidized *Xanthoceras sorbifolia* oil (AEXO) employed in this study exhibits reactivity, it suffers from high viscosity at room temperature and insufficient rigidity, necessitating the selection of suitable reactive diluents for regulation.^17^ To address this, in this paper, *Xanthoceras sorbifolia* oil dimethacrylate (MXOEA) was synthesized *via* transesterification as a reactive diluent ^18^ and blended with AEXO to construct the base resin system. Meanwhile, 2-isocyanatoethyl methacrylate (IEM) was introduced to enhance interfacial bonding through the reaction of its isocyanate groups with the hydroxyl groups of wood.^19^ The high reactivity of the isocyanate moiety enables rapid curing of the adhesive with a high crosslinking density, but this tends to reduce the toughness of the resulting composite material.^20^

To overcome the brittleness issue associated with highly crosslinked structures, the introduction of liquid rubber is one of the effective strategies for enhancing material toughness.^21^ Among various liquid rubber toughening agents, carboxyl-terminated butadiene-acrylonitrile rubber (CTBN) has garnered widespread attention due to its terminal carboxyl groups. Studies have shown that CTBN exhibits good compatibility with thermosetting resin matrices and can enhance the fracture toughness of materials through a phase separation mechanism;^22^ its terminal carboxyl groups can undergo esterification with the hydroxyl groups in the resin, forming a chemically bonded interface between the toughening phase and the matrix, thereby strengthening the interfacial adhesion between the two phases. However, while toughening the material, the rubber phase often leads to a decrease in rigidity and thermal properties. Existing studies have demonstrated that constructing a synergistic system of rigid fillers and flexible polymer networks can effectively balance the strength and toughness of materials.^23^ To this end, researchers have proposed a composite modification strategy that introduces rigid nanofillers into rubber-toughened systems to compensate for the loss of rigidity.^24^ Among these, carbon nanofibers, owing to their unique network-forming capability and excellent mechanical properties, are regarded as one of the ideal reinforcing fillers,^25–27^ demonstrating potential in enhancing the overall performance of composite materials.^28^ Based on this, the present study proposes the construction of a CTBN/nano-silicon carbide (SiC) composite toughening system in the *Xanthoceras sorbifolia* oil-based adhesive. The aim is to alleviate the brittleness of the highly crosslinked network *via* CTBN, while leveraging the rigidity-enhancing effect and interface-optimizing function of nano-SiC to maintain or even enhance the overall rigidity and interfacial stability of the adhesive alongside the improvement in toughness,^29^ thereby achieving synergistic regulation of the toughness and rigidity of the adhesive. This strategy holds significant application value for high-performance wood-based composites that are subjected to dynamic loads and require interfacial integrity.

This study aims to develop a high-performance bio-based thermosetting adhesive derived from *Xanthoceras sorbifolia* oil. First, MXOEA was synthesized as a reactive diluent *via* transesterification and acrylation reactions, and blended with AEXO to prepare the base resin system, with the introduction of 2-isocyanatoethyl methacrylate (IEM) to construct a highly crosslinked network. On this basis, a synergistic incorporation of CTBN and SiC into this system is proposed for the first time to overcome the common brittleness issue of highly crosslinked systems and achieve synergistic optimization of toughness and rigidity, a composite modification strategy that has not been previously reported in the research of vegetable oil-based adhesives. This study will systematically evaluate the processability, curing characteristics, thermomechanical behavior, and basic mechanical properties of the adhesive, and apply it to table tennis blades, a scenario demanding high mechanical performance, to verify its feasibility in actual products and achieve high-value utilization of *Xanthoceras sorbifolia* oil. The research outcomes will provide theoretical foundations and technical solutions for the development of high-performance bio-based adhesives for plywood as alternatives to petroleum-based products.

## 2. Materials and methods

### 2.1. Materials

*Xanthoceras sorbifolia* oil (XO) was purchased from Qiaodongfang Biofuel Group Co., Ltd. The following reagents were purchased from Shanghai Macklin Biochemical Technology Co., Ltd: diethanolamine (DEA, 99%), sodium methoxide (CH_3_ONa, 98%), methacrylic anhydride (MAA, 94%), 4-dimethylaminopyridine (DMAP, 99.8%), cation exchange resin IR120 (Amberlite IR120, H resin, analytical grade), triphenylphosphine (PPh_3_/C_18_H_15_P, 99.8%), hydroquinone (C_6_H_5_O_2_, 99%), 2-isocyanatoethyl methacrylate (IEM, 98%), benzoyl peroxide (BPO, 99%), and γ-glycidoxypropyltrimethoxysilane (KH-560, 97%). Glacial acetic acid (CH_3_COOH, analytical grade) and sodium chloride (NaCl, analytical grade) were supplied by Tianjin Xinbote Chemical Co., Ltd, while hydrogen peroxide (H_2_O_2_, 30%) and acrylic acid (AA, analytical grade) were purchased from Sinopharm Chemical Reagent Co., Ltd. Sodium carbonate (NaCO_3_, analytical grade) and sodium bicarbonate (NaHCO_3_, analytical grade) were obtained from Tianjin Xinbote Chemical Co., Ltd, while anhydrous ethanol (analytical grade) and oxalic acid (C_6_H_7_O_4_, analytical grade) were purchased from Tianjin Zhiyuan Chemical Reagent Co., Ltd. Nano-SiC (40 nm) was provided by Guangzhou Metallurgy and Metallurgical Group Co., Ltd, and deionized water was used throughout the experiments.

### 2.2. Preparation of CTBN-SiC compositely modified *Xanthoceras sorbifolia* oil-based adhesive

**Fig. 1:**
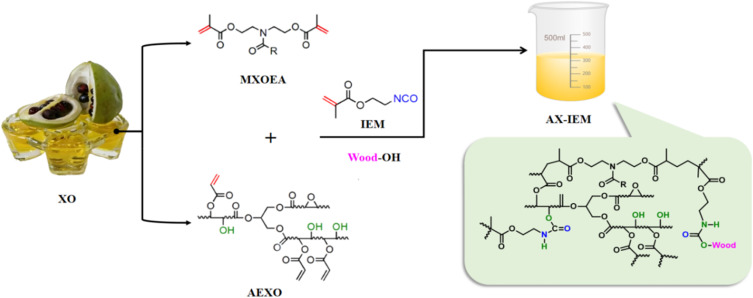
Crosslinking mechanism of AEXO, MXOEA IEM and the possible reactions between the adhesives and wood.

MXOEA and AEXO, prepared according to the method described in SI (SI S1), were mixed at a mass ratio of 7 : 3, and carboxyl-terminated butadiene-acrylonitrile rubber (CTBN) was added, followed by magnetic stirring at 90 °C and 300 rpm for 15 min. Subsequently, KH-560 modified nano-SiC, prepared according to the method described in SI (SI S2), was added to the system, and stirring was continued at 55 °C and 300 rpm for 90 min. After stirring, the mixture was placed in an ice-water bath and subjected to ultrasonic dispersion at 60 Hz for 30 min to ensure uniform dispersion of the fillers. Finally, benzoyl peroxide (BPO) at 2% and 2-isocyanatoethyl methacrylate (IEM) at 10% relative to the total mass of the adhesive were sequentially added, and the mixture was stirred at room temperature for 10 min to obtain the CTBN-SiC compositely modified *Xanthoceras sorbifolia* oil-based adhesive (Fig. 1).

### 2.3. Preparation of plywood specimens for verification

**Fig. 2:**
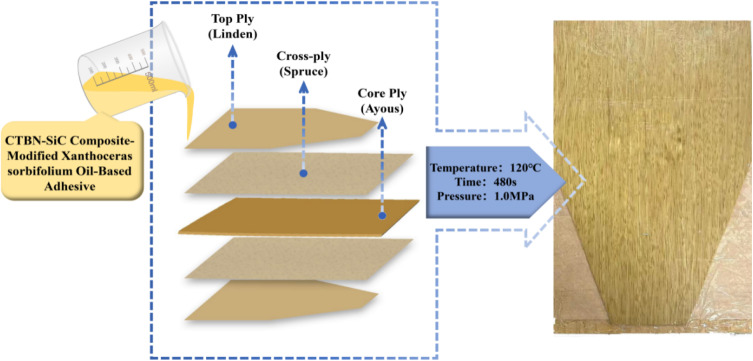
The fabrication process of plywood using the CTBN-SiC composite modified *Xanthoceras sorbifolia* oil-based adhesive, the layered wood structure of top ply (Linden)–cross ply (Spruce)–core ply (Ayous), the hot-pressing parameters, and the appearance of the finished product.

To verify the performance of the adhesive in practical application scenarios, this study selected table tennis blades, which impose stringent requirements on mechanical properties and dynamic mechanical response, as a typical target, and prepared corresponding layered plywood specimens. The fabrication process, wood assembly method, hot-pressing parameters, and appearance of the resulting plywood are shown in Fig. 2. Detailed preparation procedures are provided in SI (SI S3).

### 2.4. Methods

The chemical structure of the cured adhesive was characterized using a Fourier transform infrared spectrometer (FTIR, Nicolet iS20, Thermo Fisher Scientific, USA). Samples were prepared using the KBr pellet method, and the spectra were recorded in the range of 4000–400 cm^−1^ with a resolution of 4 cm^−1^. The microstructure was observed using a scanning electron microscope (SEM, Phenom Pro, Funa Scientific Instruments (Shanghai) Co., Ltd, China). After gold sputtering, the samples were imaged under an accelerating voltage of 10 kV using secondary electron (SE) and backscattered electron (BSE) detectors at a magnification of 2000×. The rheological properties of the liquid adhesive were measured using a rotational rheometer under a nitrogen atmosphere, with a shear rate fixed at 10 s^−1^ and the temperature ramped from 25 °C to 140 °C at a rate of °C min^−1^. Thermal stability was analyzed using a simultaneous thermal analyzer (TG-DSC, STA200, HITACHI, Japan). Samples weighing approximately 10–15 mg were heated from room temperature to 800 °C at a heating rate of 10 °C min^−1^ under a nitrogen flow of 20 mL min^−1^. Dynamic thermomechanical properties were tested using a three-point bending fixture in single cantilever mode. The cured specimen dimensions were 60 mm × 15 mm × 4 mm, with a heating rate of 5 °C min^−1^ and a test temperature range from room temperature to 160 °C. Surface wettability was characterized using a contact angle meter. A 2 µL droplet of the liquid adhesive was dispensed onto the wood surface, and the contact angle was recorded within 1 s. Each sample was tested five times, and the average value was taken. The methods for determining the mechanical properties of the plywood specimens and the bulk adhesive are provided in SI (SI S4).

## 3. Results and discussion

### 3.1. Effect of CTBN content on the mechanical properties of plywood

When the addition amount of silane coupling agent (KH-560) modified nano-SiC was 0, CTBN was added to AX-IEM10 (with an IEM addition amount of 10%), and the effects of different CTBN addition amounts on the *Xanthoceras sorbifolia* oil-based adhesive and the mechanical properties of the resulting plywood were investigated.

**Fig. 3:**
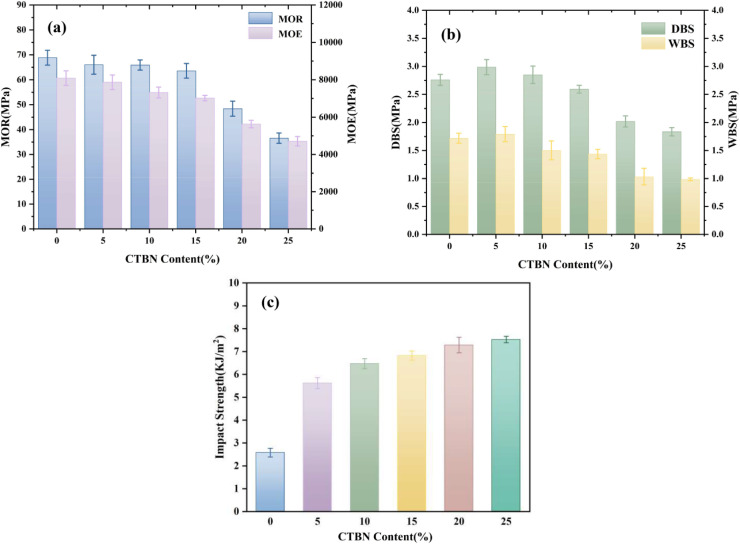
Effect of CTBN content on the mechanical properties of table tennis blades and the impact strength of the adhesive: (a) modulus of elasticity and modulus of rupture of table tennis blades; (b) dry-state and wet-state bonding strength of table tennis blades; (c) impact strength of the adhesive.

As shown in Fig. 3, with increasing CTBN content, the modulus of rupture, modulus of elasticity, and dry-state/wet-state bonding strength of the plywood exhibited a trend of initially slow decline followed by a rapid decrease. In the range of 5–15% addition, the flexible butadiene segments of CTBN were partially incorporated into the crosslinked network of the adhesive, slightly reducing the crosslinking density and enhancing the flexibility of the molecular chains. Meanwhile, the chemical bonding between the terminal carboxyl groups and the reactive groups of the adhesive, together with the interfacial interactions between the polar acrylonitrile groups and the adhesive matrix, collectively mitigated the rate of decline in mechanical properties. When the addition amount increased to 20–25%, excessive flexible segments were embedded into the crosslinked network, at which point the rubber phase became the dominant phase. Concurrently, the polarity difference between the adhesive matrix and CTBN led to macroscopic phase separation, resulting in stress concentration effects and weakened interfacial adhesion, which accelerated the degradation of mechanical properties.^30^ Meanwhile, the impact strength of the adhesive gradually increased with increasing CTBN content. This may be attributed to the flexible butadiene segments absorbing impact energy through slip deformation and inducing microcracks to delay fracture, the terminal carboxyl groups enhancing the interfacial bonding between the rubber phase and the matrix *via* chemical bonding to facilitate stress dispersion, and the acrylonitrile groups reducing the density of interfacial defects through polar interactions. This structural modification significantly enhanced toughness at the expense of some rigidity. The experimental results indicated that at a CTBN addition amount of 15%, the impact strength of the adhesive was significantly improved, while the decline in mechanical properties of the plywood was relatively small. Therefore, 15% was determined as the optimal addition amount.

### 3.2. Effect of the addition amount of silane coupling agent (KH-560) modified nano-SiC on the mechanical properties of plywood

When the CTBN addition amount was 0, KH-560 modified nano-SiC was added to AX-IEM10, and the effects of different nano-SiC addition amounts on the *Xanthoceras sorbifolia* oil-based adhesive and the mechanical properties of the resulting plywood were investigated.

**Fig. 4:**
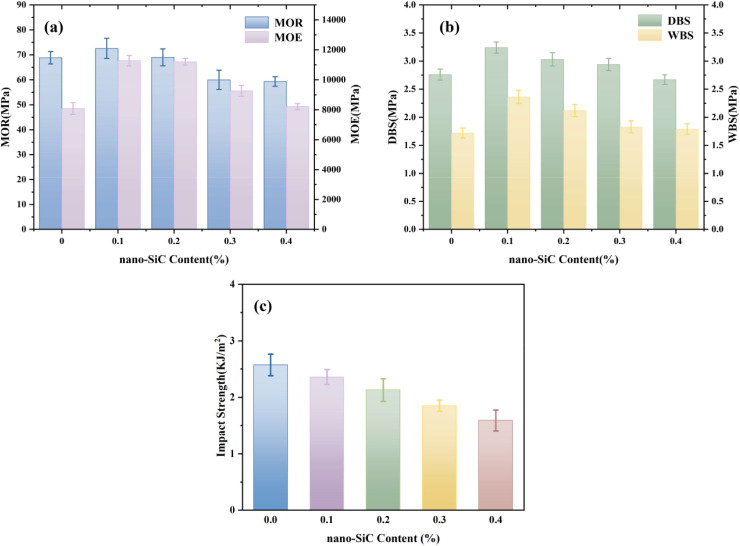
Effect of nano-SiC content on the mechanical properties of plywood and the impact strength of the adhesive: (a) modulus of elasticity and modulus of rupture of table tennis blades; (b) dry-state and wet-state bonding strength of table tennis blades; (c) impact strength of the adhesive.

As shown in Fig. 4, with increasing addition amount of silane coupling agent modified nano-SiC, the modulus of rupture, modulus of elasticity, and dry-state/wet-state bonding strength of the plywood all exhibited a trend of first increasing and then decreasing. At a nano-SiC addition amount of 0.1%, the silane groups on its surface formed strong interfacial bonding with the adhesive matrix *via* chemical bonds or hydrogen bonds. The nanoparticles restricted the movement of polymer segments, enhancing the modulus of elasticity. Meanwhile, their high specific surface area enabled adsorption of stress at crack tips, delaying crack propagation, thereby improving the modulus of rupture and bonding performance. In addition, the hydrophobicity of nano-SiC inhibited moisture absorption by the adhesive layer, reducing the disruption of interfacial hydrogen bonds under wet conditions and maintaining bonding stability. When the addition amount increased to 0.2–0.4%, excessive nano-SiC formed stress concentration points due to agglomeration effects, disrupting the continuity of the matrix and weakening load transfer efficiency, leading to a decline in the relevant properties. As the amount of KH-560 modified nano-SiC increased, the impact strength of the adhesive gradually decreased. The main reason is that the rigid nature of nano-SiC particles restricted the slippage and deformation ability of the molecular chains in the adhesive matrix, reducing energy dissipation efficiency and making the material more prone to brittle fracture under impact. Although an appropriate amount of nano-SiC could enhance strength, it led to deterioration in impact performance. The experimental results indicated that at an addition amount of 0.1%, the mechanical properties of the plywood reached their optimum, while the reduction in impact strength was relatively small.

### 3.3. Effect of CTBN-SiC content on the mechanical properties of plywood

When the CTBN addition amount was 15%, silane coupling agent (KH-560) modified nano-SiC was added to AX-IEM10, and the effects of different SiC addition amounts on the *Xanthoceras sorbifolia* oil-based adhesive and the mechanical properties of the resulting plywood were investigated.

**Fig. 5:**
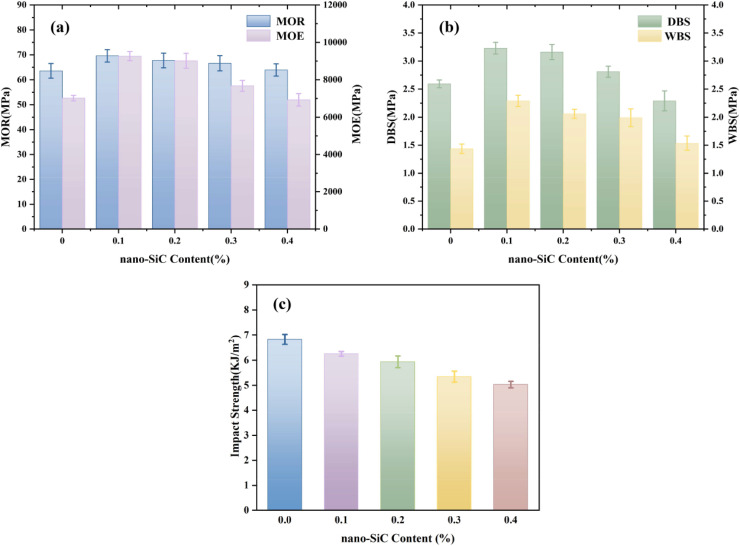
Effect of nano-SiC content on the mechanical properties of plywood and the impact strength of the adhesive: (a) modulus of elasticity and modulus of rupture of table tennis blades; (b) dry-state and wet-state bonding strength of table tennis blades; (c) impact strength of the adhesive.

As shown in Fig. 5, when the CTBN addition amount was 15%, with increasing nano-SiC content, the modulus of rupture, modulus of elasticity, and dry-state/wet-state bonding strength of the plywood all exhibited a trend of first increasing and then decreasing. At a nano-SiC addition amount of 0.1%, KH-560 enhanced the filler-matrix interfacial bonding through chemical linkages, and the uniformly dispersed nano-SiC increased the modulus of elasticity of the plywood and delayed crack propagation by adsorbing stress at crack tips, thereby improving the modulus of rupture and bonding performance. The hydrophobic nature of nano-SiC acted synergistically with the flexible buffering effect of CTBN to suppress the disruption of interfacial hydrogen bonds under wet conditions. When the nano-SiC addition amount increased to 0.2–0.4%, the excessive filler formed stress concentration points due to agglomeration, disrupting the continuity of the matrix and reducing load transfer efficiency. The agglomerates also hindered the formation of the crosslinked network between CTBN and the matrix, weakening the cohesive strength of the adhesive layer. As the addition amount of KH-560 modified nano-SiC increased, the impact strength of the adhesive gradually decreased. This may be attributed to the fact that while nano-SiC enhanced rigidity, it excessively suppressed the toughness imparted by CTBN, disrupting the balance between energy dissipation and load-bearing capacity of the material, leading to a transition in impact failure mode from ductile fracture to brittle fracture. Experimental results indicated a synergistic optimization effect between CTBN and silane-modified nano-SiC. When the CTBN addition amount was 15%, and the silane-modified nano-SiC addition amount was 0.1% (based on the total mass of the CTBN-modified adhesive), the plywood achieved the best overall mechanical properties with the minimal reduction in impact strength.

### 3.4. FTIR analysis of *Xanthoceras sorbifolia* oil-based adhesive

To elucidate the curing reaction characteristics of the base crosslinked network, Fourier transform infrared (FTIR) spectroscopy was performed on MXOEA, AX (without IEM), and AX-IEM10 (with an IEM addition amount of 10%). The results indicated that the double bonds in MXOEA were efficiently converted during the curing process *via* free radical polymerization, and IEM was successfully incorporated into the crosslinked network through chemical bonding (SI S5).

**Fig. 6:**
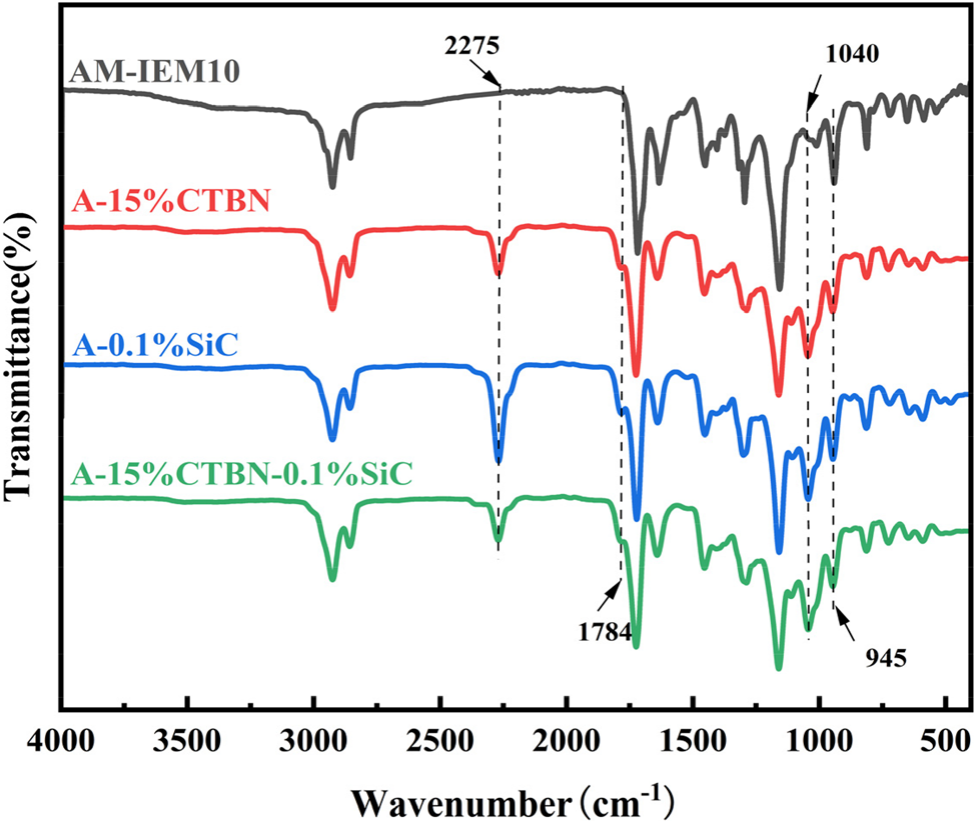
FTIR spectra of the *Xanthoceras sorbifolia* oil-based adhesive.

On this basis, further FTIR characterization was performed on AX-IEM10, A-15%CTBN (AX-IEM10 with 15% CTBN), A-0.1% SiC (AX-IEM10 with 0.1% nano-SiC), and A-15% CTBN-0.1% SiC (AX-IEM10 with 15% CTBN and 0.1% nano-SiC), and the results are shown in Fig. 6. Compared with AX-IEM10, all modified samples exhibited a new absorption peak at 2275 cm^−1^: in A-15%CTBN and A-15%CTBN-0.1%SiC, this peak was attributed to the stretching vibration of the nitrile (C≡N) segments in the CTBN backbone;^31^ whereas in A-0.1%SiC and A-15%CTBN-0.1%SiC, this peak likely originated from the adsorption of reaction by-products or residual unreacted coupling agent due to the high specific surface area of nano-SiC, with A-0.1%SiC showing a more pronounced peak intensity owing to the enrichment effect of nanoparticle adsorption. The appearance of a new absorption peak at 1784 cm^−1^ indicated that in A-15%CTBN, the carboxyl groups of CTBN underwent esterification with the hydroxyl/epoxy groups of the matrix to form ester groups (C=O),^32^ whereas in A-0.1%SiC and the composite sample, the silanol groups (Si–OH) generated from the hydrolysis of KH-560 formed Si–O–C bonds with the matrix carboxyl groups,^33^ accompanied by the formation of ester linkages. The enhanced absorption peak at 1040 cm^−1^ corresponded to the vibrational characteristics of Si–O–Si and Si–O–C bonds formed by the hydrolysis and condensation of KH-560, confirming the formation of stable chemical bonding between the silane coupling agent, nano-SiC, and the matrix. Furthermore, the decrease in the intensity of the characteristic epoxy peak at 945 cm^−1^ was attributed to the consumption of epoxy groups in the system by both the esterification reaction with CTBN carboxyl groups and the bonding between KH-560 silanol groups and epoxy groups.

### 3.5. SEM analysis of *Xanthoceras sorbifolia* oil-based adhesive

**Fig. 7:**
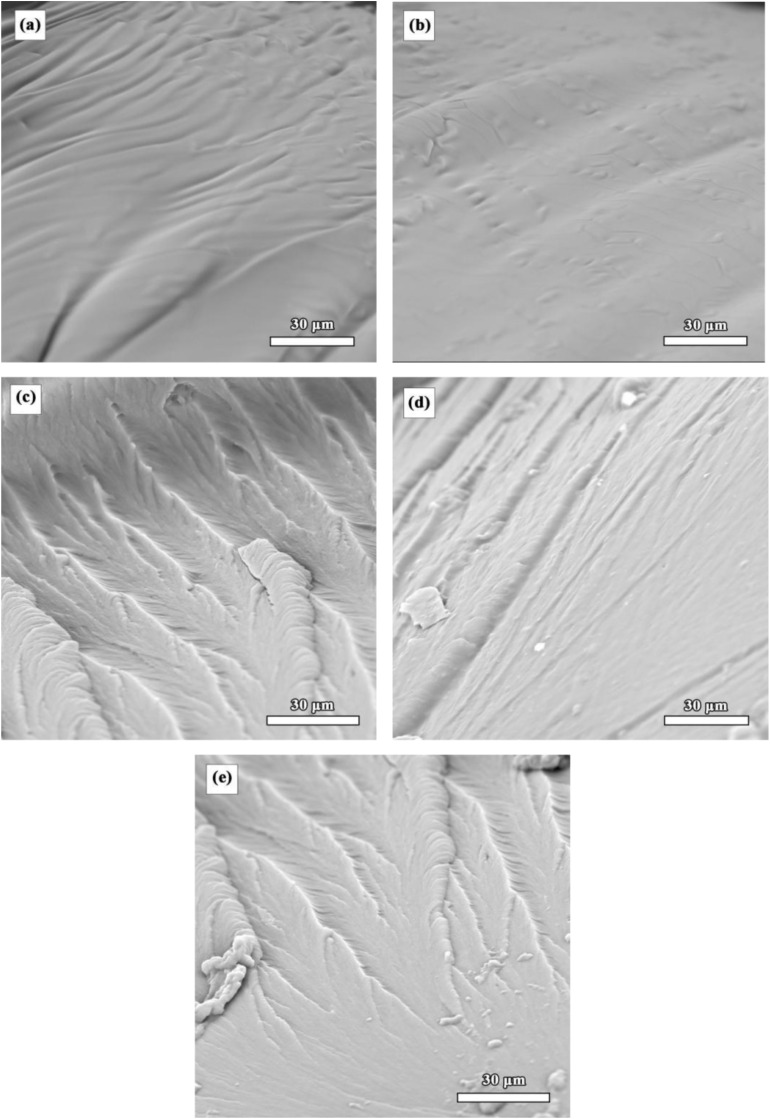
SEM images of *Xanthoceras sorbifolia* oil-based adhesive: (a) AX; (b) AX-IEM10; (c) A-15%CTBN; (d) A-0.1%SiC; (e) A-15% CTBN-0.1%SiC.

The fracture morphology of the *Xanthoceras sorbifolia* oil-based adhesive was characterized using scanning electron microscopy (SEM), and the results are shown in Fig. 7. As observed in Fig. 7a, the SEM image of AX shows an irregular gully-like morphology on the fracture surface, indicating plastic deformation resulting from the flexible long aliphatic chains in the vegetable oil.^34,35^ In contrast, the fracture surface of AX-IEM10 is smooth and flat (Fig. 7b), with straight crack propagation paths and no obvious branching, exhibiting typical brittle fracture characteristics.^2^ This reflects the increase in crosslinking density of the thermosetting material after the introduction of IEM. Upon the addition of 15% CTBN (Fig. 7c), the fracture surface became considerably rough, indicating ductile failure.^36^ CTBN was uniformly dispersed in the resin matrix, and these rubber particles were responsible for and contributed to a faster transfer of the applied energy through the polymer matrix rather than concentrating it at specific points, thereby providing better flexibility and toughness to the blended system.^37^Fig. 7d shows that the SiC particles in A-0.1%SiC were uniformly distributed within the resin matrix, with no obvious agglomeration. The fracture surface was relatively smooth and dense, which was attributed to the good interfacial bonding between the KH-560 modified SiC and the resin matrix, with the interfacial strength enhanced through Si–O–Si and Si–O–C chemical bonds.^38^ The nanoparticles hindered crack propagation through the crack pinning mechanism,^39^ improving the fracture toughness of the thermosetting resin. In the CTBN/SiC composite system (Fig. 7e), both micron-sized CTBN domains and nano-SiC particles were present. Crack deflection from the original path can be observed. When the crack deviates from its original path, the energy required for crack propagation and sample failure increases;^40^ consequently, the impact strength of the CTBN/SiC compositely modified adhesive was higher than that of the unmodified adhesive. This composite toughening approach enhances the toughness of the adhesive while maintaining the rigidity of the matrix, achieving an effective balance between toughness and rigidity.

### 3.6. Rheological analysis of the *Xanthoceras sorbifolia* oil-based adhesive

**Fig. 8:**
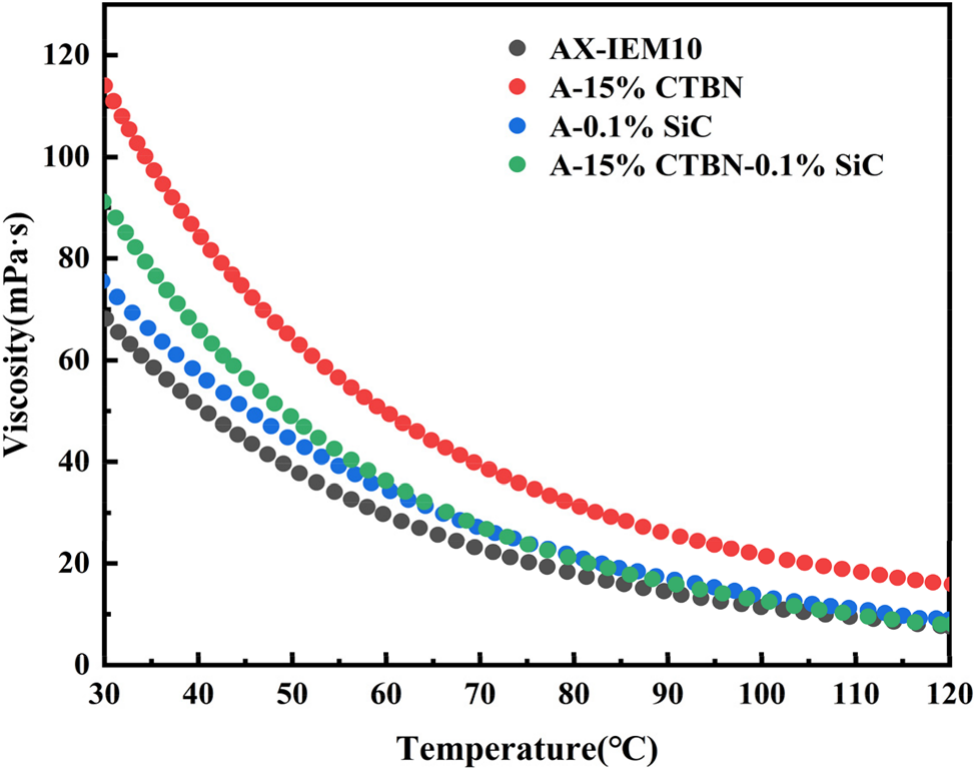
Viscosity *versus* temperature curves of the *Xanthoceras sorbifolia* oil-based adhesive.

Acrylate resins typically exhibit high viscosity at room temperature, which may adversely affect the applicability for the gluing process. To address this, the viscosity-temperature characteristics of the prepared *Xanthoceras sorbifolia* oil-based adhesive systems were investigated, and the results are shown in Fig. 8. The viscosity of all adhesives decreased exponentially with increasing temperature. At 30 °C, the viscosity of AX-IEM10 was 68.2 mPa s, that of A-15%CTBN increased to 113.98 mPa s, that of A-0.1%SiC was 72.44 mPa s, and that of A-15%CTBN-0.1%SiC was 88.02 mPa s. The viscosity of all adhesives at 30 °C was below 500 mPa s, meeting the processability requirements for plywood adhesives.^34^ As the temperature increased, the viscosity of each system further decreased, indicating that this adhesive system possesses good thermal fluidity, facilitating coating and wood penetration in practical applications.

The viscosity of A-15%CTBN was significantly higher than that of AX-IEM10, which was attributed to the physical entanglement of the long-chain structure of CTBN and the three-dimensional network formed by chemical crosslinking between its carboxyl groups and the matrix, enhancing intermolecular friction. The viscosity of A-0.1%SiC increased slightly compared with that of AX-IEM10, which was due to the rigid confinement effect of the nanoparticles slightly increasing the viscosity, while silane bonding suppressed filler agglomeration, avoiding a sharp rise in viscosity. The viscosity of A-15%CTBN-0.1%SiC fell between the two, exhibiting an intermediate value, indicating a synergistic regulation mechanism between CTBN and SiC: on one hand, the uniformly dispersed SiC nanoparticles reduced excessive entanglement of CTBN molecular chains through steric hindrance effects, decreasing the physical crosslinking density between chain segments; on the other hand, the flexible CTBN chain segments partially alleviated the rigid confinement imposed by SiC particles on the molecular motion of the matrix through molecular slippage. These two effects counterbalanced each other, resulting in a moderate viscosity of the composite system under the dual actions of viscosity increase from CTBN and viscosity reduction from SiC. The viscosity of A-15%CTBN-0.1%SiC fell between the two, indicating the presence of a synergistic regulation mechanism between CTBN and SiC. The flexible CTBN network alleviated the rigid confinement of SiC particles through molecular chain slippage, while the uniform dispersion of SiC reduced the entanglement density of CTBN chain segments. This viscosity variation pattern reveals the regulatory mechanism of the synergistic effect between CTBN and nano-SiC on the rheological behavior of the adhesive.

### 3.7. Thermal stability analysis of *Xanthoceras sorbifolia* oil-based adhesive

**Fig. 9:**
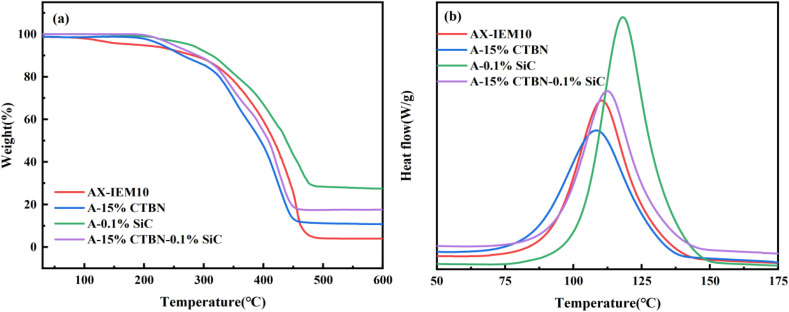
Thermogravimetric (TG) and derivative thermogravimetric (DTG) curves of the *Xanthoceras sorbifolia* oil-based adhesive: (a) TG curves; (b) DSC curves.

The thermal stability of the four adhesives was characterized using thermogravimetric analysis (TG) (Fig. 9a). All adhesives exhibited similar thermal decomposition patterns, but distinct differences in thermal decomposition behavior were observed among the different adhesives. AX-IEM10 showed no significant weight loss in the range of 0–269 °C, indicating good thermal stability. In the range of 269–496 °C, it underwent three-stage decomposition: initial cleavage of ester bonds and decomposition of fatty acid chains (269–320 °C), thermal pyrolysis of the ester backbone (320–420 °C), and oxidative carbonization of the carbon chains (420–496 °C).^17^ After the introduction of CTBN, the initial decomposition temperature of the adhesive decreased to 191.62 °C. This is attributed to the poor thermal stability of CTBN, whose molecular chains begin to undergo scission at relatively low temperatures. A-0.1%SiC exhibited the optimal thermal stability, which was attributed to the incorporation of rigid SiC that suppressed the mobility of polymer chains by forming constrained regions,^25^ effectively hindering the diffusion and escape of volatile small-molecule products generated from thermal decomposition. In the composite system, the modification effects of CTBN and SiC balanced each other. The introduction of CTBN slightly reduced the initial decomposition temperature of the system, but its molecular chain energy dissipation mechanism^40,41^ delayed the thermal decomposition process; the physical barrier effect of SiC significantly increased the char residue. The synergistic effect of the two enabled the compositely modified system to maintain good thermal stability, addressing the issue of poor thermal stability caused by the addition of CTBN. Therefore, the present study demonstrates that the appropriate combination of CTBN and SiC with the *Xanthoceras sorbifolia* oil-based adhesive can ensure that the adhesive retains its thermal stability while undergoing toughening modification. Furthermore, the initial decomposition temperatures of all adhesives shown in the figure were well above the hot-pressing temperature of plywood, indicating that their thermal stability meets practical requirements.^42^

The curing process of the liquid *Xanthoceras sorbifolia* oil-based adhesive was monitored by DSC. As shown in Fig. 9b, the exothermic peaks in the curves indicate the free radical polymerization process of the resin 2. The onset curing temperature of AX-IEM10 was 81.45 °C upon the addition of 15% CTBN (A-15%CTBN), the onset curing temperature decreased to 75.48 °C, and the intensity of the exothermic peak weakened. This was attributed to the flexible segments of CTBN increasing the free volume of the system and reducing its viscosity, allowing the curing reaction to be initiated at a lower temperature. In contrast, after the addition of 0.1% SiC (A-0.1%SiC), the onset curing temperature significantly increased to 92.11 °C, and the exothermic peak became sharper and more intense. This may be due to the physical barrier effect of the nanoparticles, which increased the difficulty for reactive groups to encounter each other, or because the addition of nanoparticles slightly increased the viscosity of the system,^43^ delaying the initiation of the reaction. Notably, in the CTBN/SiC compositely modified system (A-15%CTBN-0.1%SiC), the onset curing temperature was 80.49 °C, which was close to that of the unmodified system. This indicates that the effects of CTBN and SiC on the curing behavior balanced each other, forming an effective competitive balance mechanism. The tendency of CTBN to promote chain segment motion and the tendency of SiC to constrain chain segment motion counteracted each other, bringing the curing behavior of the composite system back to the baseline level. This characteristic offers significant processing advantages: while meeting the requirements for toughness and rigidity, there is no need to adjust the original curing process parameters, and the existing hot-pressing conditions can be directly applied, providing benefits in terms of process adaptability and economic efficiency.

### 3.8. Dynamic thermomechanical analysis of *Xanthoceras sorbifolia* Bunge seed oil-based adhesive

**Fig. 10:**
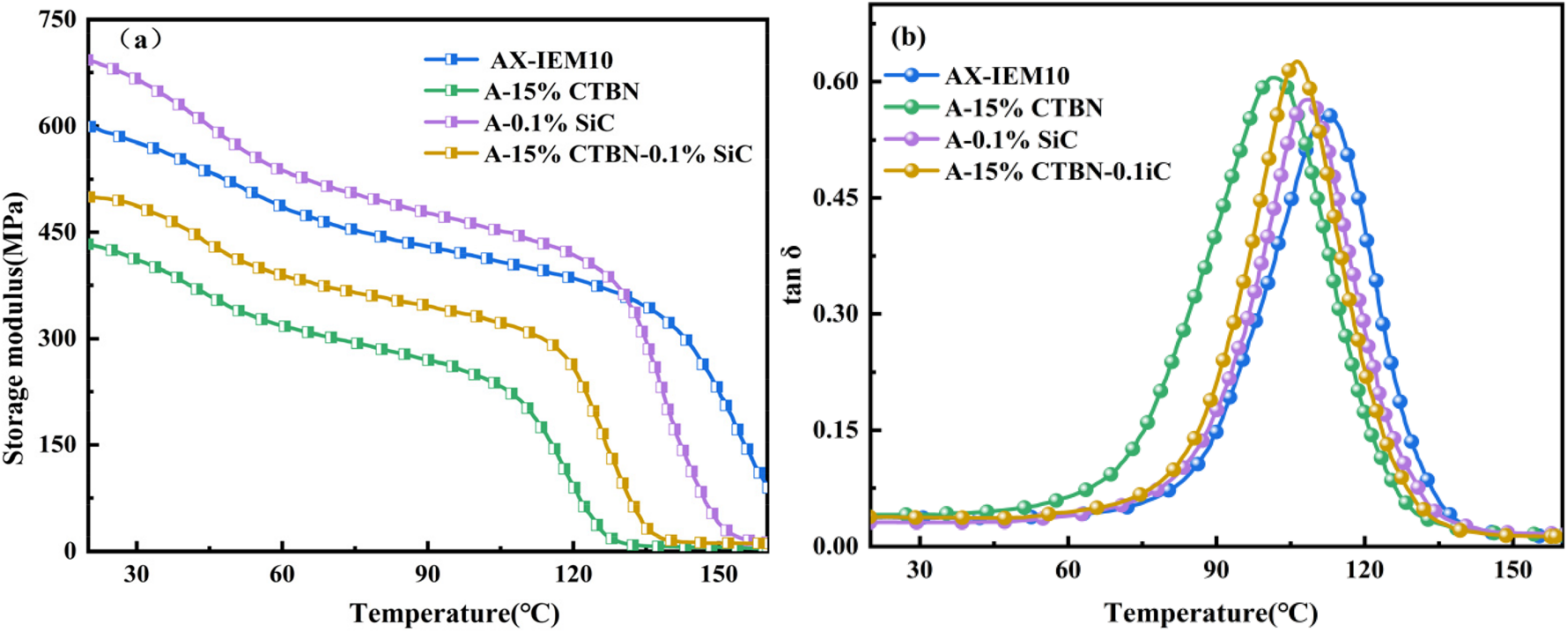
DMA curves and loss factor curves of the *Xanthoceras sorbifolia* oil-based adhesive: (a) DMA curves; (b) loss factor curves.

Dynamic mechanical analysis (DMA) of the four *Xanthoceras sorbifolia* oil-based adhesives revealed the correlation between their thermomechanical properties and modification mechanisms. As shown in Fig. 10a, A-0.1%SiC exhibited the highest initial storage modulus (*E*′) due to the enhanced interfacial bonding between the incorporated nano-SiC and the matrix. However, at high temperatures (>130 °C), the modulus rapidly decreased because the high thermal conductivity of SiC accelerated segmental relaxation and interfacial debonding induced by differential thermal expansion.^43^ AX-IEM10, relying on its high crosslinking density, exhibited a relatively high initial modulus and a gradual decrease at elevated temperatures. A-15%CTBN showed the lowest initial modulus due to the plasticizing effect of the flexible segments, which significantly reduced the crosslinking density; at high temperatures, the softening of the rubber phase further weakened the mechanical properties. The storage modulus of A-15%CTBN-0.1%SiC was intermediate: at low temperatures, SiC enhanced rigidity, while at high temperatures, the competition between the plasticizing effect of CTBN and the confinement effect of SiC slowed the decrease in modulus. As shown in Fig. 10b, loss factor analysis revealed that AM-IEM10 had the highest glass transition temperature (*T*_g_) at 112 °C; however, its homogeneous structure limited energy dissipation capacity,^44^ resulting in the lowest tan *δ* peak value. The *T*_g_ of A-15%CTBN decreased to 103 °C, and its microphase-separated structure contributed to energy dissipation through interfacial friction, yielding the second-highest tan *δ* peak value. For A-0.1%SiC, interfacial confinement increased *T*_g_ to 108 °C, but the high thermal conductivity suppressed segmental motion, leading to the lowest tan *δ* peak value. A-15%CTBN-0.1%SiC exhibited a *T*_g_ of 107 °C and the highest tan *δ* peak value through the synergistic effect of interfacial friction from CTBN phase separation and vibration from SiC, demonstrating the advantage of multiscale energy dissipation. These results indicate that single modifications dominate performance through chemical crosslinking or physical plasticization, whereas composite modification requires balancing the interfacial synergy between rigid fillers and flexible toughening phases.^45^

### 3.9. Impact strength analysis of *Xanthoceras sorbifolia* oil-based adhesive

**Fig. 11:**
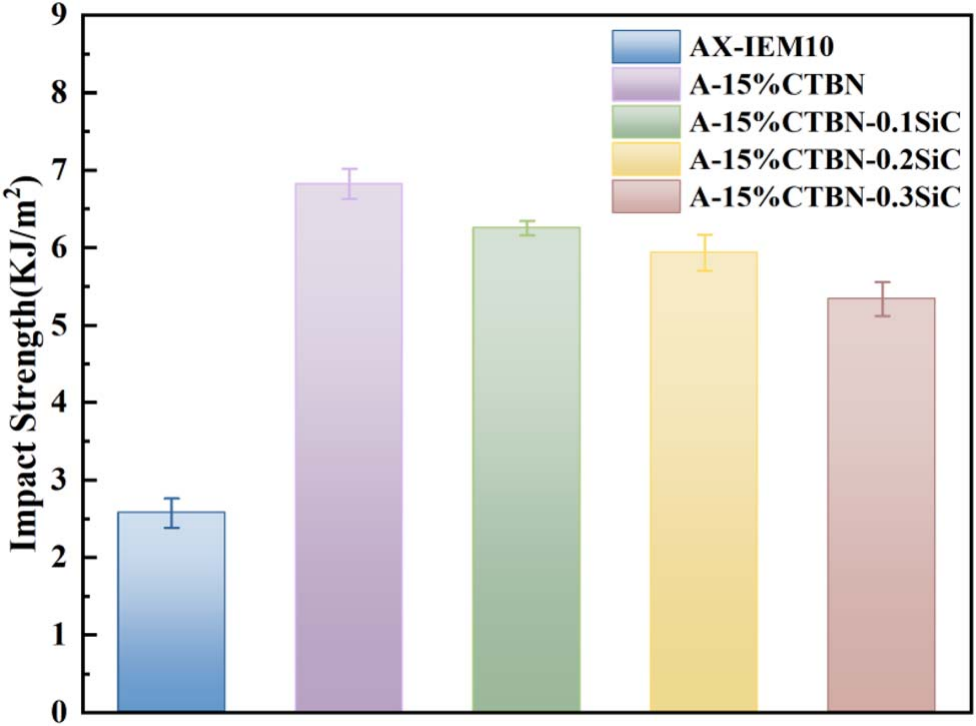
Impact strength of *Xanthoceras sorbifolia* oil-based adhesive.

The impact properties of AX-IEM10, A-15%CTBN, A-15%CTBN-0.1%SiC, A-15%CTBN-0.2%SiC, and A-15%CTBN-0.3%SiC were investigated using a pendulum impact test. As shown in Fig. 11, the experimental data indicated that the impact strength of AX-IEM10 was 2.575 kJ m^−2^, while after the introduction of 15% CTBN, the impact strength of A-15%CTBN significantly increased to 6.824 kJ m^−2^. This was mainly attributed to the CTBN rubber phase absorbing impact energy through cavitation and shear yielding mechanisms,^46,47^ leading to an enhancement in the impact strength of the adhesive. When 0.1% nano-SiC was incorporated, the impact strength of A-15%CTBN-0.1%SiC slightly decreased to 6.25 kJ m^−2^, indicating that although the uniform dispersion of SiC could hinder crack propagation through crack pinning and deflection mechanisms, its introduction might slightly suppress the toughening efficiency of the CTBN phase. When the nano-SiC content increased to 0.2% and 0.3%, the impact strength further decreased, which was mainly attributed to stress concentration caused by nanoparticle agglomeration and the restriction of plastic deformation of the CTBN phase by excessive SiC, resulting in reduced material toughness. In summary, the introduction of CTBN significantly improved the impact strength of the *Xanthoceras sorbifolia* oil-based adhesive, while the introduction of nano-SiC appropriately weakened the toughening effect. However, the impact strength of the CTBN-SiC compositely modified *Xanthoceras sorbifolia* oil-based adhesive remained much higher than that of AX-IEM10.

### 3.10. Surface wettability analysis of *Xanthoceras sorbifolia* oil-based adhesive

**Fig. 12:**
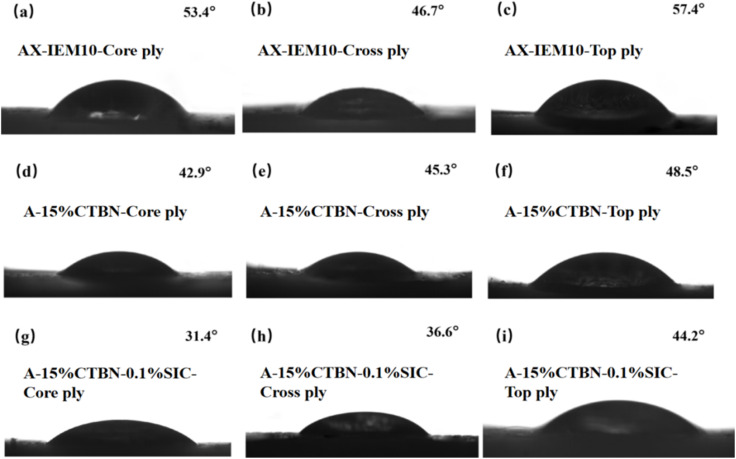
Contact angles between *Xanthoceras sorbifolia* Bunge seed oil-based adhesive and various wood veneers. (a)–(c) AX-IEM10 on core ply, Cross ply, and Top ply, respectively; (d)–(f) A-15%CTBN on core ply, Cross ply, and Top ply, respectively; (g)–(i) A-15%CTBN-0.1%SIC on core ply, Cross ply, and Top ply, respectively.

Contact angle measurements revealed the differences in surface wetting behavior of three adhesives, namely AX-IEM10, A-15%CTBN, and A-15%CTBN-0.1%SiC, on the core ply (Ayous wood), cross ply (Spruce wood), and top ply (Linden wood). As shown in Fig. 12, the contact angle measurements indicated that A-15%CTBN exhibited contact angles of 42.9°, 45.3°, and 48.5°on the core ply (Ayous wood), cross ply (Spruce wood), and top ply (Linden wood), respectively, which were significantly lower than those of AX-IEM10. This was attributed to the polar interactions between the carboxyl groups (–COOH) of CTBN and the hydroxyl groups (–OH) of wood,^48^ as well as its elastomeric characteristics promoting interfacial spreading. The contact angles of A-15%CTBN-0.1%SiC on the various wood species further decreased to 31.4°, 36.6°, and 44.2°. This performance improvement may originate from two synergistic mechanisms: on one hand, the uniform dispersion of nano-SiC formed a micro/nano structure that enhanced surface roughness, promoting capillary penetration of the adhesive; on the other hand, the polar interactions between nano-SiC and the carboxyl groups of CTBN, combined with the elastomeric characteristics, generated a synergistic effect,^49^ ultimately achieving comprehensive optimization of wetting performance. These results indicate that A-15%CTBN synergistically improved the wettability of the adhesive on wood through polar groups and elastomeric characteristics, while the synergistic mechanism of A-15%CTBN-0.1%SiC significantly optimized the spreading and penetration behavior of the adhesive on the wood surface, enhancing the interfacial bonding between the adhesive and the wood veneers.

### 3.11. Photographs of fabricated table tennis blades

**Fig. 13:**
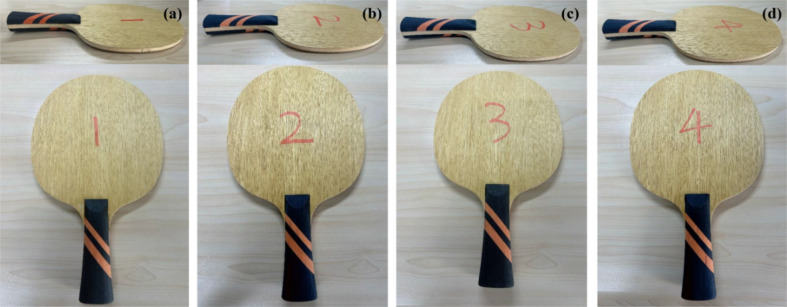
Photographs of fabricated table tennis blades: (a) blade pressed with AX; (b) blade pressed with AX-IEM10; (c) blade pressed with A-15%CTBN; (d) blade pressed with A-15%CTBN-0.1%SiC.

To demonstrate the forming state of the developed adhesive in actual products, the laminated plywood prepared with different adhesive formulations was further processed into a typical laminated structural product—table tennis blades. As shown in Fig. 13, the finished blades corresponding to all adhesive formulations exhibited regular shapes, smooth surfaces, and no visible interlayer defects, preliminarily indicating that this adhesive system possesses good structural integrity and formability in complex wood components.

## 4. Conclusions

In this study, a formaldehyde-free, high-performance bio-based thermosetting adhesive was prepared using *Xanthoceras sorbifolia* oil as the raw material. By introducing CTBN and SiC to construct a composite toughening network, the brittleness issue of the adhesive was addressed, and a balance between its flexibility and rigidity was achieved. Fourier transform infrared (FTIR) spectroscopy confirmed that the carboxyl groups of CTBN underwent esterification with the matrix, while SiC formed chemical bonding with the matrix through Si–O–Si and Si–O–C bonds. Scanning electron microscopy (SEM) revealed that IEM imparted brittle fracture characteristics to the adhesive, whereas after the composite modification with CTBN and SiC, the synergistic effect between the micron-sized CTBN domains and nano-SiC particles enabled the adhesive to achieve an effective balance between toughness and rigidity. Thermal analysis results indicated that the introduction of SiC effectively compensated for the negative impact of CTBN on thermal stability, and the onset of curing temperature of the composite system was essentially consistent with that of the unmodified system, achieving a balance between thermal stability and processability. Dynamic mechanical analysis (DMA) and surface wettability tests further revealed that the compositely modified adhesive achieved synergistic regulation of toughness and rigidity through a multiscale energy dissipation mechanism, while the synergistic effect between CTBN and SiC significantly improved the wettability of the adhesive on wood surfaces, which was beneficial for enhancing interfacial bonding quality. When the addition amounts of CTBN and nano-SiC were 15% and 0.1%, respectively, the impact strength of the adhesive increased by 142.7% compared with that of the unmodified system. Moreover, the plywood specimens prepared with this adhesive exhibited modulus of rupture, modulus of elasticity, and dry-state/wet-state bonding strength that met the key mechanical and water-resistance requirements for high-performance wood-based composites. Further application validation in table tennis blades preliminarily demonstrated that this adhesive system possesses good structural integrity and formability in complex wood components. This study provides an effective design and preparation strategy for developing high-performance bio-based adhesives for plywood as alternatives to petroleum-based products, showing promising application prospects.

## Conflicts of interest

The authors declare that they have no conflict of interest.

## Supplementary Material

RA-016-D5RA09970F-s001

## Data Availability

Data will be made available on request. Supplementary information (SI) is available. See DOI: https://doi.org/10.1039/d5ra09970f.
