# Supplementary material for: Preparation and performance study of CTBN/nano-SiC composite toughened Xanthoceras sorbifolia oil-based adhesive
Source: RSC Adv. 2026 May 14;16(26):24006–20. doi: 10.1039/d5ra09970f (PMC13182513; doi:10.1039/d5ra09970f)
Supplement: RA-016-D5RA09970F-s001 [file RA-016-D5RA09970F-s001.pdf]

Supplementary Information (SI) for RSC Advances.
This journal is © The Royal Society of Chemistry 2026

Supplementary Information

Preparation and Performance Study of CTBN/Nano-SiC Composite Toughened Xanthoceras sorbifolia Oil-based Adhesive

Rao Duan [a,b], Jie Wang [c], Yihua Zhang [c], Zhenpeng Wang [a,b], Lei Jiao [a,b], Yan Long [a,b], Tao Hou [a,b], Gaole Zhao [a,b,], and Yinan Hao [a,b,]*

[a] College of Material Science and Art Design, Inner Mongolia Agricultural University, Hohhot 010018, China

[b] National Forestry Grassland Engineering Technology Research Center for Efficient Development and Utilization of Sandy Shrubs, Hohhot 010018, China

[c] Tianjin 729 Sports Goods Co., Ltd., Tianjin 300392, China

* Corresponding author.

mail address: nanyihao83@163.com

Total number of pages: 9

Total number of figures: 1

Total number of tables: 0

Contents

S1 Synthesis Procedures of MXOEA and AEXO

S2 Preparation of Silane Coupling Agent-Modified Nano-SiC

S3 Detailed Process Parameters for Plywood Preparation

S4 Methods for Determining Mechanical Properties of Plywood Specimens and Adhesive

S5 FTIR spectra of MXOEA, AX, and AX-IEM10

S1 Synthesis Procedures of MXOEA and AEXO

S1.1 Synthesis Procedure of MXOEA

Under a nitrogen atmosphere, 105 g of Xanthoceras sorbifolia oil (XO) was mixed with 70 g of diethanolamine and 1.62 g of sodium methoxide as a catalyst. The mixture was stirred at 80℃ for 20 min, then heated to 120℃ and reacted at 520 rpm for 4 h. After cooling, the Xanthoceras sorbifolia oil-based diol (XOEA) was obtained with a mass yield of 98.62% (based on XO). Subsequently, XOEA and methacrylic anhydride (MAA) were reacted at a mass ratio of 1:2, using 4-dimethylaminopyridine (DMAP, 2% by mass of XOEA) as a catalyst, at 65℃ and 520 rpm for 5 h. After the reaction, the product was washed successively with saturated $NaHCO_3$ solution and saturated NaCl solution, and then subjected to rotary evaporation to obtain Xanthoceras sorbifolia oil dimethacrylate (MXOEA) with a mass yield of 81.40% (based on XOEA).

S1.2 Synthesis Procedure of AEXO

50 g of XO, 1.5 g of cation exchange resin, and 45 mL of glacial acetic acid were mixed uniformly. 125 mL of 30% H_2O_2 solution was slowly added dropwise at room temperature. The mixture was then heated to 65℃ and stirred continuously at 500 rpm for 3 h to obtain epoxidized Xanthoceras sorbifolia oil (EXO). The product was washed successively with saturated Na_2CO_3 solution and saturated NaCl solution until neutral, and then purified by rotary evaporation, yielding a mass yield of 89.50% (based on XO). Subsequently, 100 g of the obtained EXO was mixed with 3.75 g of triphenylphosphine (PPh_3) and 1.25 g of hydroquinone as a polymerization inhibitor. 50 g of acrylic acid (AA) was slowly added dropwise at 80℃, followed by heating to 100℃ and reaction at 500 rpm for 4 h to obtain acrylated epoxidized Xanthoceras sorbifolia oil (AEXO). The reaction product was likewise washed with saturated Na_2CO_3 solution and saturated NaCl solution until neutral, and purified by rotary evaporation, yielding a mass yield of 83.46% (based on EXO).

S2 Preparation Process of Silane Coupling Agent Modified Nano-SiC

Interfacial interactions exist between nanoparticles and the organic resin matrix. The use of coupling agents can enhance the bonding between them, ensuring good adhesion between the filler and the matrix, and improving bonding strength and durability. Silane coupling agents commonly used in resins include γ-aminopropyltriethoxysilane (KH-550) and γ-glycidoxypropyltrimethoxysilane (KH-560). Compared with KH-550, KH-560 possesses long-chain methylene groups and flexible ether bonds, which can effectively resist external stress and disperse stress concentration. This helps to alleviate point-like and local stresses in the bonding area, improving overall bonding strength and durability. Therefore, KH-560 was used to modify SiC in this study.

S2.1 Raw Material Pretreatment

The nano-SiC powder (average particle size of 40 nm) was placed in a vacuum drying oven and dried at 80°C for 12 h to remove adsorbed moisture.

S2.2 Preparation of KH-560 Hydrolyzate

An appropriate amount of anhydrous ethanol was taken, and γ-glycidoxypropyltrimethoxysilane (KH-560, 97%) was added at 2% of the total system mass. The pH was adjusted to 4-5 using oxalic acid ($C_6H_7O_4$), and the mixture was allowed to stand at room temperature for 30 min to complete the activation of the coupling agent, yielding a transparent hydrolyzate.

S2.3 Surface Modification of Nano-SiC

The dried nano-SiC powder was weighed and dispersed in anhydrous ethanol, followed by ultrasonic treatment at 60 Hz in an ice-water bath for 1 h to achieve thorough dispersion of the particles. The above hydrolyzate was slowly added dropwise to the ethanol di

spersion of nano-SiC. After the addition was completed, the mixture was transferred to a constant-temperature oil bath at 70°C and reacted under mechanical stirring at 300 rpm for 3 h.

S2.4 Post-Reaction Treatment

After the reaction, the mixture was cooled to room temperature and centrifuged at 8000 rpm for 15 min to collect the precipitate. The precipitate was washed three times with anhydrous ethanol, with centrifugation performed after each wash. The washed product was dried in a vacuum drying oven at 50°C for 12 h to obtain KH-560 modified nano-SiC material, which was then sealed and stored for further use.

S3 Detailed Processing Parameters for Plywood Preparation

S3.1 Veneer Materials

The plywood adopted a five-layer structure, with the top ply made of Linden wood, the cross ply made of Spruce wood, and the core ply made of Ayous wood.

S3.2 Adhesive Application and Assembly

The prepared CTBN-SiC composite modified Xanthoceras sorbifolia oil-based adhesive was placed in a vacuum oven for degassing to remove internal bubbles and residual volatile substances. The adhesive was uniformly applied onto the surface of the veneers, with the adhesive application amount controlled at 180 g/m² (per single side). Assembly was carried out with perpendicular grain orientation, where the grain direction of the top ply was perpendicular to that of the cross ply, and the grain direction of the cross ply was perpendicular to that of the core ply.

S3.3 Aging and Hot Pressing

After assembly, the mat was subjected to open aging at room temperature for 15 min, and then placed in a hot press for hot pressing at 120°C and 1.0 MPa for 480 s.

S3.4 Cooling and Post-Treatment

After hot pressing, the mat was immediately wrapped with plastic wrap and slowly cooled in an insulation chamber to prevent warping or cracking caused by a sudden temperature drop. After cooling to room temperature, the cured mat was processed into standard test specimens and finished blade shapes.

S4 Methods for Determining the Mechanical Properties of Plywood Specimens and Bulk Adhesive

S4.1 Determination of Modulus of Rupture and Modulus of Elasticity

The modulus of rupture and modulus of elasticity of the plywood specimens were determined using a microcomputer-controlled electronic universal testing machine (WDW-20A, Tianchen Machine Manufacturing Co., Ltd., China). The specimen length was set to (20t ± 50) mm based on the thickness t, the width was 50 mm, the span was 20t mm, and the loading rate was 20 mm/min.

S4.2 Determination of Impact Strength

The impact strength of the bulk adhesive was determined using a Charpy impact tester, and the specimen dimensions were 80 mm × 10 mm × 4 mm.

S4.3 Determination of Dry-State and Wet-State Bonding Strength

The dry-state and wet-state bonding strength tests were conducted in accordance with relevant standards: dry-state specimens were tested directly; wet-state specimens were immersed in water at 63°C for 3 h, then removed and cooled for 10 min before testing. The specimen dimensions were uniformly 100 mm × 25 mm, and the loading rate was 5 mm/min.

S4.4 Application Performance Validation

To comprehensively evaluate the performance of the adhesive in practical application scenarios, the pressed laminated plywood specimens were further processed into application components with a specific structure (using table tennis blades as prototypes components) to demonstrate the forming state of the developed adhesive in actual products.

S5 FTIR spectra of MXOEA, AX, and AX-IEM10.

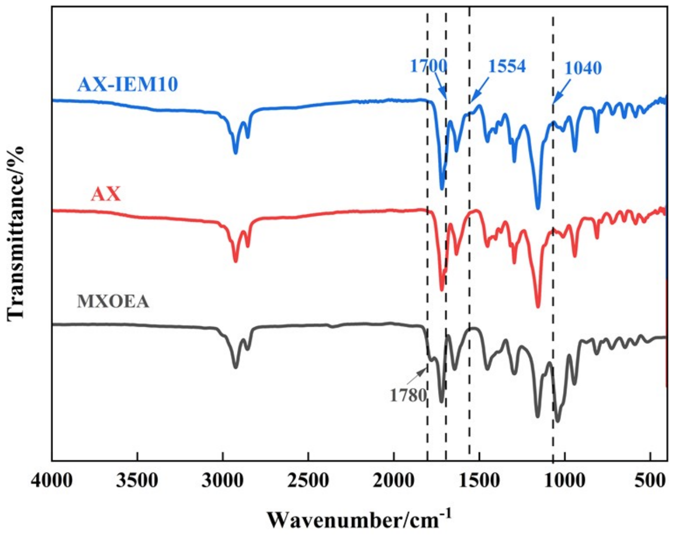

Fig. 1 FTIR spectra of MXOEA, AX, and AX-IEM10.

Fourier transform infrared (FTIR) spectroscopy was performed on MXOEA, adhesive AX (Xanthoceras sorbifolia oil-based adhesive without IEM), and AX-IEM10 (Xanthoceras sorbifolia oil-based adhesive with 10% IEM), and the results are shown in Fig. 1. MXOEA exhibited a stretching vibration peak of the methacrylate carbonyl group (C=O) at 1782 cm^{-1} and a symmetric stretching vibration peak of the ester bond (C-O-C) at 1040 cm^{-1}, indicating the successful introduction of diester groups into its structure. In the FTIR spectrum of AX, the carbonyl peak of MXOEA shifted to 1720 cm^{-1} and overlapped with the ester peak of AEXO, while the characteristic ester peak at 1040 cm^{-1} disappeared due to the formation of the crosslinked network, indicating that the reactive double bonds in MXOEA were fully converted during the curing process via free radical polymerization, forming a crosslinked structure with AEXO. Compared with AX, AX-IEM10 exhibited a new peak at 1700 cm^{-1} attributed to the urethane bond (-NH-CO-O-), and a peak at 1554 cm^{-1} corresponding to the N-H bending vibration of the urethane group [1], confirming that the isocyanate groups (-NCO) of IEM reacted with the hydroxyl groups of AEXO. Furthermore, no characteristic peak of -NCO was observed around 2270 cm^{-1}, indicating that the isocyanate groups were fully involved in the reaction. These results demonstrate that IEM was successfully incorporated into the crosslinked network through chemical bonding, and the double bonds in MXOEA were efficiently converted during the curing process via free radical polymerization.

[1] Liu, W., Xie, T., & Qiu, R. (2016). Improvement of properties for biobased composites from modified soybean oil and hemp fibers: Dual role of diisocyanate. Composites Part A: Applied Science and Manufacturing, 90, 278-285
